# The associations between the polymorphisms in the *CTLA-4* gene and the risk of Graves’ disease in the Chinese population

**DOI:** 10.1186/1471-2350-14-46

**Published:** 2013-04-19

**Authors:** Liang Du, Jiqiao Yang, Jichong Huang, Yaxian Ma, Haichuan Wang, Tianyuan Xiong, Zhangpeng Xiang, Yonggang Zhang, Jin Huang

**Affiliations:** 1 West China Medical School/West China Hospital, Sichuan University, Chengdu, Sichuan 610041, China; 2 The Periodical Press of West China Hospital, Sichuan University, Guoxuexiang 37, Chengdu, Sichuan 610041, China

**Keywords:** Graves’ disease, *CTLA-4*, Polymorphism, Chinese, Meta-analysis

## Abstract

**Background:**

The associations between the polymorphisms in Cytotoxic T lymphocyte-associated molecule-4 (*CTLA-4*) gene and Graves’ disease (GD) have been extensively investigated in Chinese population. However, the results were inconsistent. The objective of this study is to investigate the associations between the polymorphisms in *CTLA-4* gene and the risk of GD by meta-analysis.

**Methods:**

We searched Pubmed database, Medline (Ovid) database, CNKI database and Wanfang database, covering all studies until August 11, 2012. Statistical analysis was performed by using the Revman4.2 software and the Stata10.0 software.

**Results:**

A total of 28 case–control studies concerning the most widely studied three polymorphisms [+49A/G(rs231775), -318C/T(rs5742909) and CT60(rs3087243)] for Chinese population in 21 publications were included. The results suggested that the G allele carriers (GG+GA) might have an increased risk of GD when compared with the AA homozygote carriers for the +49A/G polymorphism (GG+GA vs. AA: OR = 2.57, 95%CI = 1.87-3.52). However, as to the -318C/T polymorphism and CT60 polymorphism, the results indicated that the variant allele carriers might have decreased risks of GD when compared with the homozygote carriers (−318C/T: TT+TC vs. CC: OR = 0.78, 95%CI = 0.62-0.97; CT60: AA+AG vs. GG: OR = 0.64, 95%CI = 0.52-0.78).

**Conclusions:**

The current meta-analysis indicated that the polymorphisms in the *CLTA-4* gene might be risk factors for GD in the Chinese population. In future, more large-scale case–control studies are needed to validate these results.

## Background

Graves’ disease (GD) is a complex autoimmune thyroid disease, which is caused by excessive production of thyroid hormone and characterized by an enlarged thyroid gland, protrusion of the eyeballs, a rapid heartbeat and nervous excitability [1]. It is reported that GD occurs in about 1.2% in Western population and 0.25–1.09% in Chinese population [2]. It is widely accepted that GD is caused by complex interactions between many genetic factors and environmental factors. Numerous studies have been published focusing on the topic of genetic factors of GD risk in the Chinese population. Many genes involved in the inception and evolution of GD have been identified as GD candidate genes, such as *ADRB2*[3], *TSHR*[4], *CTLA-4*[5] and *IL-13* gene [6]. And among them, the *CTLA-4* gene is one of the most extensively studied.

Cytotoxic T lymphocyte-associated molecule-4 (*CTLA-4*) is a T cell surface molecule [7]. It is a negative regulator of T cell activation and plays an important role in the pathogenesis of GD. The *CTLA-4* gene is localized on chromosome 2q33. Many polymorphisms have been identified in the *CTLA-4* gene. It is reported that the polymorphisms in *CTLA-4* gene might influence the expression of the protein, and might play important roles in the pathogenesis of GD [8]. Up to now, many studies have been performed to investigate the associations between the polymorphisms in the *CTLA-4* gene and the risk of GD. Among them, the +49A/G, -318C/T and CT60 polymorphisms were the most widely studied. To this day, the associations between polymorphisms of the *CTLA-4* gene and the risk of GD have been widely investigated in the Chinese population. However, the results were inconsistent, and the associations were not yet formally evaluated. In order to derive a more precise conclusion, we performed a meta-analysis to assess the associations between the polymorphisms in the *CTLA-4* gene and the risk of GD in the Chinese population. To our knowledge, this is the first comprehensive genetic meta-analysis performed in the Chinese population for Graves’ disease.

## Methods

### Study identification and selection

A literature search in Pubmed database, Medline (Ovid) database, CNKI database and Wanfang database was carried out to identify studies investigating the association between the Graves’ disease risk and the *CTLA-4* polymorphisms on Aug 11th, 2012. The search terms were as follows: *Graves’ disease* or *GD* in combination with *polymorphism* or *variant* or *mutation* and in combination with *CTLA-4* or Cytotoxic T lymphocyte-associated molecule-4*.* All languages were included. The inclusion criteria were: (a) studies evaluating the association between the (+49A/G, -318C/T and CT60) polymorphisms in the *CTLA-4* gene and Graves’ disease risk in the Chinese population, (b) the design should be a case–control design, (c) sufficient data (genotype distributions of cases and controls) available to calculate an odds ratio (OR) with its 95%CI (confidence interval), (d) genotype distributions in control group should be consistent with Hardy-Weinberg equilibrium (HWE). Studies were excluded if one of the following existed: (a) the studied populations were based on family or sibling pairs, (b) genotype frequencies or numbers were not presented in the original studies, (c) reviews and abstracts. If more than one study was published by the same authors using the same case series or overlapping case series, studies with the largest size of samples were included.

### Data extraction

Two investigators independently extracted the data and reached a consensus on all items. The following items were extracted from each study if available: first author’s name, publication year, province of origin, age of cases, genotype number in cases and controls and genotyping method.

### Statistical analysis

The strength of associations between the polymorphisms in the *CTLA-4* gene and Graves’ disease risk was assessed by odds ratios (OR) with the corresponding 95% confidence intervals (CI). The genetic models evaluated for the pooled OR of the polymorphisms were dominant models (GG+GA vs. AA for the +49A/G, TT+TC vs. CC for the -318C/T, and AA+AG vs. GG for the CT60). OR was analyzed by a fixed-effects model (the Mantel-Haenszel method) or a random-effects model (the DerSimonian and Laird method) according to the heterogeneity. Heterogeneity was assessed by a *X*^*2*^ based *Q* statistic and was considered statistically significant at *p*-value <0.10. When the *P* value was more than 0.10, the pooled OR was calculated by the fixed-effects model, otherwise, a random-effects model was used. The significance of the pooled OR was determined by the *Z*-test and *p*-value less than 0.05 was considered as statistically significant. Sensitivity analysis was conducted by sequential excluding a single study each time in an attempt to identify the potential influence of the individual data set to the pooled ORs. In addition, the possible publication bias was investigated with the Begg’s funnel plot. Funnel plot asymmetry was assessed by Egger’s linear regression test [9]. For each polymorphism, other genetic models were also used to assess the association with the risk of Graves’ disease (for the +49A/G polymorphism: GG vs. AA+GA, GG vs. AA, GA vs. AA, G vs. A; for the -318C/T polymorphism: TT vs. CC+TC, TT vs. CC, TC vs. CC, T vs. C; for the CT60 polymorphism: AA vs. AG+GG, AA vs. GG, AG vs. GG, A vs. G). HWE was tested by Person’s *X*^*2*^ test. Statistical analysis was performed using Revman4.2 software and Stata10.0 software.

## Results

### Studies selection and characteristics

The selection process of studies was as follows. Briefly, a total of 429 results were identified after an initial search from the Pubmed, Medline (Ovid), CNKI and Wanfang databases. After reading the titles and abstracts, 302 results were excluded for being irrelevant to *CTLA-4* polymorphisms and Graves’ disease risk, abstracts, reviews or duplications of search results. After reading full-texts of the remaining 127 studies, 68 studies were excluded for not relevant to the GD risk in the Chinese population, and 9 studies were excluded for not relevant to the investigated polymorphisms (+49A/G, -318C/T and CT60). Thus, 50 studies were left for data extraction. And then, a total of 54 case–control studies were extracted for these three polymorphisms. Among 54 case–control studies, genotype numbers for control group in 7 studies were not consistent with HWE, data in 19 studies were overlapped. So these 27 case–control studies were excluded. Finally, a total of 28 case–control studies in 21 publications were identified for meta-analysis [2,10-27]. Summary of the properties of the studies are listed in Table 1. Overall, there were 17 case-controls studies for the +49A/G polymorphism [2,5,11-13,15,17-25,27,28], 7 case–control studies for the -318C/T polymorphism [2,10-12,14,26,28] and 4 case–control studies for the CT60 polymorphism [2,10,16,18]. The genotype distributions for these polymorphisms are listed in Table 2.

**Table 1:** Properties of the 21 case–control studies included in meta-analysis

**Author**	**Publication year**	**Province**	**Case age(year)**	**Case number**	**Control number**	**Genotyping method**	**Polymorphisms**
Chong, K K [10]	2008	Hong Kong	<16	177	151	PCR-RFLP	−318C/T, CT60
Du, Y T [11]	2005	Tianjin	-	96	60	PCR-PFLP	+49A/G, -318C/T
Guo, Z Q [12]	2010	Shandong	44.17 ± 1.54	102	100	PCR-PFLP	+49A/G, -318C/T
Han, S Z [2]	2006	Chongqing	-	263	196	PCR-PFLP	+49A/G, -318C/T, CT60
Jiang, B R [13]	2005	Shandong	43.8 ± 13.5	98	95	PCR-PFLP	+49A/G
Kang, Y Z [14]	2010	Ningxia	43.7 ± 11.5	61	60	PCR-PFLP	−318C/T
Shen, F X [15]	2005	Zhejiang	36.0 ± 12.3	107	57	PCR-PFLP	+49A/G
Tsai, S T [16]	2008	Taiwan	10.2 ± 3.3	189	620	PCR-RFLP	CT60
Wang, L [17]	2001	Shandong	40 ± 13	87	84	PCR-PFLP	+49A/G
Wang, P W [18]	2007	Taiwan	39 ± 13	208	192	PCR-RFLP	+49A/G, CT60
Wang, Q H [19]	2003	Zhejiang	45.7 ± 9.5	64	28	PCR-PFLP	+49A/G
Wang, S Q [20]	2010	Shandong	41.5 ± 28.5	90	90	PCR-PFLP	+49A/G
Weng, Y C [21]	2005	Taiwan	34.0 ± 11.8	107	101	PCR-PFLP	+49A/G
Yang, J [22]	2012	Xi’an	34.14 ± 12.23	303	215	PCR-PFLP	+49A/G
Yao, B [23]	2005	Guangdong	36.6 ± 12.8	120	123	PCR-PFLP, PCR-SSLP	+49A/G
Yu, Q L [24]	2006	Guangdong	45 ± 11	100	100	PCR-PFLP	+49A/G
Yu, Z Y [25]	2008	Xi’an	36.7 ± 13.28	125	126	PCR-RFLP	+49A/G
Zhang, H [26]	2010	Shandong	-	211	85	PCR-PFLP	−318C/T
Zhang, J L [27]	2008	Shandong	37.8 ± 13.3	186	100	PCR-PFLP	+49A/G
Zhang, Q [28]	2006	Zhejiang	-	89	60	PCR-RFLP	+49A/G, -318C/T
Zhao, S X [5]	2010	Shandong, Suzhou, Guangdong, Fujian	-	2640	2204	Mass-Array™	+49A/G

**Table 2:** **Distribution of *CTLA-4 *genotype among patients with Graves’ disease and controls included in the meta-analysis**

**Polymorphism**	**Author**	**Case**			**Control**			**Case**		**Control**		**HWE**
+49A/G polymorphism		AA	AG	GG	AA	AG	GG	A	G	A	G	
	Du, Y T [11]	1	27	68	7	26	27	29	163	40	80	Yes
	Guo, Z Q [12]	24	52	26	41	47	12	100	104	129	71	Yes
	Han, S Z [2]	33	95	135	32	89	75	161	365	153	239	Yes
	Jiang, B R [13]	10	44	44	33	46	16	64	132	112	78	Yes
	Shen, F X [15]	5	34	68	4	30	23	44	170	38	76	Yes
	Wang, L [17]	3	47	37	32	42	10	53	121	106	62	Yes
	Wang, P W [18]	15	69	124	18	77	97	99	317	113	271	Yes
	Wang, Q H [19]	21	24	19	12	15	1	66	62	39	17	Yes
	Weng, Y C [21]	8	53	46	15	58	28	69	145	88	114	Yes
	Wang, S Q [20]	5	47	38	24	52	14	57	123	100	80	Yes
	Yang, J [22]	12	139	152	29	97	89	163	443	155	275	Yes
	Yao, B [23]	9	53	58	11	57	55	71	169	79	167	Yes
	Yu, Q L [24]	13	36	51	28	46	26	62	138	102	98	Yes
	Yu, Z Y [25]	13	45	67	20	60	46	71	179	100	152	Yes
	Zhang, J L [27]	16	100	70	32	43	25	132	240	107	93	Yes
	Zhang, Q [28]	2	29	58	7	26	27	33	145	40	80	Yes
	Zhao, S X [5]	104	730	1030	156	823	945	938	2790	1135	2713	Yes
−318C/T polymorphism		CC	CT	TT	CC	CT	TT	C	T	C	T	
	Chong, K K [10]	147	28	2	122	29	0	322	32	273	29	Yes
	Du, Y T [11]	80	13	3	46	12	2	173	19	104	16	Yes
	Guo, Z Q [12]	84	18	0	76	23	1	186	18	175	25	Yes
	Kang, Y Z [14]	52	8	1	48	11	1	112	10	107	13	Yes
	Zhang, H [26]	175	35	1	69	16	0	385	37	154	16	Yes
	Han, S Z [2]	159	98	2	103	85	2	416	26	291	101	Yes
	Zhang, Q [28]	65	22	6	46	12	8	152	110	104	16	Yes
CT60 polymorphism		GG	AG	AA	GG	AG	AA	G	A	G	A	
	Chong, K K [10]	125	48	4	88	51	12	298	56	227	75	Yes
	Han, S Z [2]	184	71	8	123	60	13	439	87	306	86	Yes
	Tsai, S T [16]	136	48	5	372	216	32	320	58	960	280	Yes
	Wang, P W [18]	138	46	5	125	58	9	322	56	308	76	Yes

### Quantitative synthesis

#### The +49A/G polymorphism

**Figure 1:**
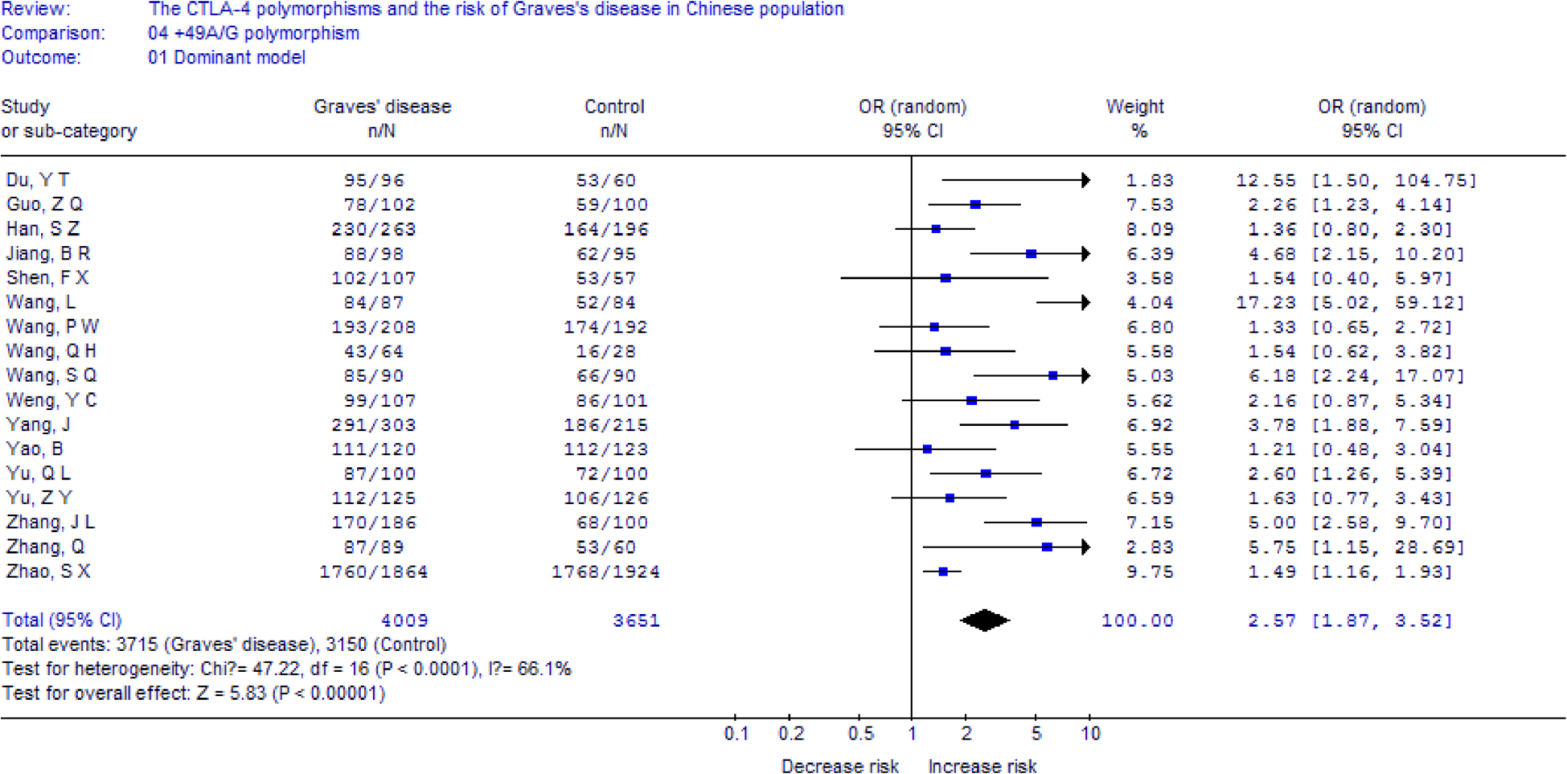
**Meta-analysis with a random-effects model for the association between GD risk and the *CTLA-4 *+49A/G polymorphism (GG+GA vs. AA).**

A total of 4009 cases and 3651 controls from 17 case–control studies were included for data synthesis. As is shown in Figure 1, we analyzed the heterogeneity of GG+GA vs. AA for all 17 studies and the value of *X*^*2*^ was 47.22 with 16 degrees of freedom and *p*-value < 0.00001 in a random-effects model. Additionally, I-square value is another index of the test of heterogeneity. In Figure 1, the I-square was 66.1%, suggesting a moderate of heterogeneity. Thus, we chose the random-effects model to synthesize the data. Overall, OR was 2.57 (95%CI = 1.87-3.52) and the test for overall effect *Z* value was 5.83 (*p*-value < 0.00001). The results suggested that the G allele carriers might have an increased risk of Graves’ disease compared with those individuals with the AA homozygote. Statistically similar results were obtained after sequential excluding each case–control study for the GG+GA vs. AA comparative, suggesting the stability of our meta-analysis. Significant publication bias was detected in the funnel plot (figure not shown), and in the Egger’s test, the result was: t = 2.82, *p*-value = 0.013, which also indicated considerable publication bias. Summary of the results of other genetic comparisons are listed in Table 3.

**Table 3:** Summary of different comparative results

**Polymorphism**	**Genetic model**	**Participants**	**OR (95%CI)**	***Z***	***p*-value**	**I**^**2**^**, %**	***P***_**Het**_	**Effect model**
+49A/G	GG+GA vs. AA	7660	2.57(1.87,3.52)	5.83	< 0.00001	66.1	< 0.0001	Random
	GG vs. GA+AA	7660	2.11(1.70,2.63)	6.69	< 0.00001	70.3	< 0.00001	Random
	GG vs. AA	4402	3.87(2.59,5.80)	6.57	< 0.00001	74.3	< 0.00001	Random
	GA vs. AA	4053	1.96(1.44,2.67)	4.25	< 0.00001	60.8	0.0006	Random
	G vs. A	15320	1.88(1.58,2.23)	7.18	< 0.00001	76.8	< 0.00001	Random
−318C/T	TT+TC vs. CC	1701	0.78(0.62,0.97)	2.18	0.03	0	0.86	Fixed
	TT vs. TC+CC	1701	0.76(0.37,1.53)	0.78	0.44	0	0.92	Fixed
	TT vs. CC	1291	0.70(0.35,1.43)	0.97	0.33	0	0.90	Fixed
	TC vs. CC	1672	0.78(0.62,0.98)	2.11	0.03	0	0.86	Fixed
	T vs. C	3402	0.80(0.66,0.98)	2.12	0.03	0	0.88	Fixed
CT60 G/A	AA + AG vs. GG	1977	0.64(0.52,0.78)	4.34	< 0.0001	0	0.82	Fixed
	AA vs. AG + GG	1977	0.43(0.26,0.72)	3.22	0.001	0	0.82	Fixed
	AA vs. GG	1379	0.39(0.23,0.65)	3.62	0.0003	0	0.81	Fixed
	AG vs. GG	1889	0.69(0.55,0.85)	3.52	0.0004	0	0.82	Fixed
	A vs. G	3954	0.65(0.54,0.77)	4.9	< 0.00001	0	0.81	Fixed

#### The -318C/T polymorphism

**Figure 2:**
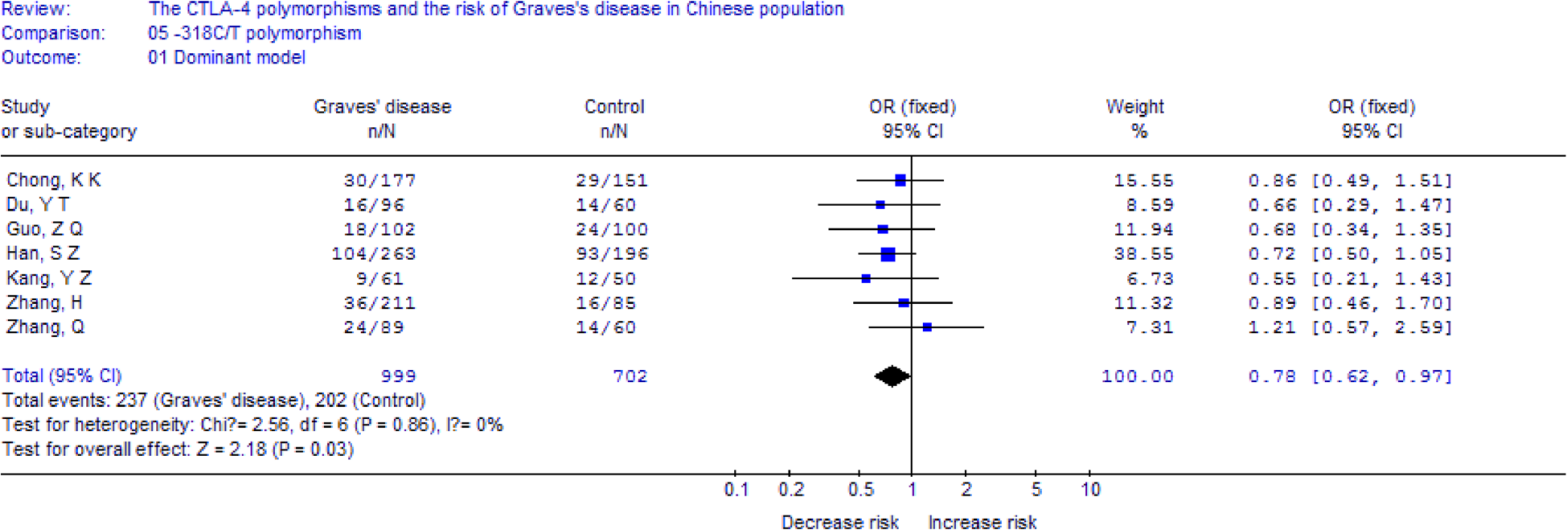
**Meta-analysis with a random-effects model for the association between GD risk and the *CTLA-4 *-318C/T polymorphism (TT+TC vs. CC).**

A total of 999 cases and 702 controls from 7 case–control studies were included for data synthesis. As is shown in Figure 2, we analyzed the heterogeneity of TT+TC vs. CC for all 7 studies and the value of *X*^*2*^ was 2.56 with 6 degrees of freedom and *p*-value = −0.86 in a fixed-effects model. Additionally, I-square value is another index of the test of heterogeneity. In Figure 2, the I-square was 0%, suggesting an absent of heterogeneity. Thus, we chose the fixed-effects model to synthesize the data. Overall, OR was 0.78 (95%CI = 0.62-0.97) and the test for overall effect *Z* value was 2.18 (*p*-value = 0.03). The results suggested that the T allele carriers might have a decreased risk of Graves’ disease compared with those individuals with the CC homozygote. Statistically similar results were obtained after sequential excluding each case–control study for the TT+TC vs. CC comparative, suggesting the stability of our meta-analysis. No publication bias was detected with either the funnel plot (figure not shown) or Egger’s test (t = 0.09, *p*-value = 0.929). Summary of the results of other genetic comparisons are listed in Table 3.

#### The CT60 polymorphism

**Figure 3:**
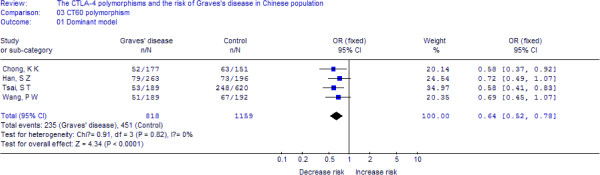
**Meta-analysis with a random-effects model for the association between GD risk and the *CTLA-4 *CT60 polymorphism (AA+AG vs. GG).**

A total of 818 cases and 1159 controls from 4 case–control studies were included for data synthesis. As is shown in Figure 3, we analyzed the heterogeneity of AA+AG vs. GG for all 4 studies and the value of *X*^*2*^ was 0.91 with 3 degrees of freedom and *p*-value = 0.82 in a fixed-effects model. Additionally, I-square value is another index of the test of heterogeneity. In Figure 3, the I-square was 0%, suggesting an absent of heterogeneity. Thus, we chose the fixed-effects model to synthesize the data. Overall, OR was 0.64 (95%CI = 0.52-0.78) and the test for overall effect *Z* value was 4.34 (*p*-value = 0.001). The results suggested that the A allele carriers might have a decreased risk of Graves’ disease compared with those individuals with the GG homozygote. Statistically similar results were obtained after sequential excluding each case–control study for the AA+AG vs. GG comparative, suggesting the stability of our meta-analysis. No publication bias was detected with either the funnel plot (figure not shown) or Egger’s test (t = 0.19, *p*-value = 0.864). Summary of the results of other genetic comparisons are listed in Table 3.

## Discussion

Graves' disease (GD) is a thyroid-specific autoimmune disease affecting 0.25–1.09% of the Chinese population [2]. To this day, the mechanisms of GD have been widely studied from the environmental factors to the genetic factors [29]. However, the results are inconsistent and the exact mechanisms are still unrevealed. Among genetic risk factors, the *cytotoxic T lymphocyte associated-4* (*CTLA-4*) gene is one of the widely investigated. *CTLA-4* gene, which encodes a vital negative regulatory molecule of the immune system [30], has been demonstrated as candidate gene of GD [31,32]. To date, three polymorphisms (+49A/G, -318C/T and CT60) have been suggested as GD risk factors in the Chinese population. However, the results were inconsistent. Therefore, we performed a comprehensive meta-analysis to assess the association and to get more conclusive results.

This meta-analysis, including a total of 28 case–control studies in 21 publications, investigated three most widely studied polymorphisms in the *CTLA-4* gene. We found that the +49A/G polymorphism was associated with an increased risk of GD in the Chinese population, and the G allele carriers might have a higher risk of disease than the AA homozygote carriers. The results suggested a significant association between this polymorphism in the Chinese population, which is consistent with some other populations, such as the UK population [33] and the Iranian population [34]. Our results indicated that the increase in the risk is more evident in the Chinese population than in other populations, suggesting possible roles of ethnic differences in genetic backgrounds and the environment. In addition, the +49A/G polymorphism is located in exon 1, and results in a threonine-to-alanine conversion at codon 17 in the peptide leader sequence of the CTLA-4 protein. It reported that this polymorphism was associated with lower mRNA levels of the soluble alternative splice form of CTLA-4 [35]. Thus, our results could be partly explained that the variant carriers might have lower mRNA levels of the protein of the CTLA-4, and then have increased risk of the disease. In future, more studies should be performed in the Chinese population to validate these results.

A total of 999 cases and 702 controls from 7 case–control studies were included for the -318C/T polymorphism. The results suggested that the T allele carriers might be associated with a decreased risk of GD compared with CC homozygote carriers. As for the CT60 polymorphism, 818 cases and 1159 controls from 4 case–control studies were included, and the results also indicated a decrease in the risk of GD. Considering the included case–control studies for both polymorphisms were relatively small, larger number of relevant studies are needed in future to validate these results.

Hitherto, many studies have already been published focusing on the genetic risk factors of the GD among the Chinese population. For instance, Chu reported that a non-synonymous single-nucleotide polymorphism rs40401 (P27S) of the *interleukin 3* (*IL3*) gene was associated with increased risk of GD [36]; Guo found the rs568408 polymorphism in the *interleukin-12* (*IL-12*) gene was also associated with increased risk of GD [37]. In addition, polymorphisms in the *ADRB2* gene [3], *interleukin-10* (*IL-10*) gene [38], *TNF-α* gene [39] were also found to be associated with GD in Chinese population. These genes were all suggested as the candidate genes for GD in Chinese population. In future, the associations between these polymorphisms and the GD risk in Chinese population are needed to be validated by more case–control studies.

In the present meta-analysis, sensitivity analysis was performed and stability of the results was guaranteed. Publication bias was assessed by Begg’s funnel plot and Egger’s test [40]. No significant publication bias was found for the -318C/T and the CT60 polymorphism analysis, suggesting the results of these two polymorphisms were more reliable. However, we found significant publication bias for the +49A/G polymorphism. The reason might be that some reports were not published, especially for those with negative results. The results might affect the strength of the association, thus, large scale case–control studies are needed to assess the association between the +49A/G polymorphism and GD risk.

We have to mention the heterogeneity. We found significant heterogeneity for the +49A/G polymorphism. Since all participants were Chinese, the genetic background might not be taken as a factor for the heterogeneity for +49A/G polymorphism. However, some other factors, such as gender, age and location might affect the heterogeneity. In addition, we found no heterogeneity for the -318C/T and the CT60 polymorphisms, which suggested that the association for these two polymorphisms are more reliable than the +49A/G polymorphism.

It is reported that GD occurs more frequently but less severe in women than in men. In China, the different condition of disease in men and women might be similar to the situation of the world. In our study, the data was not analyzed by gender because of the lack of original information for these populations. In future, such subgroup studies are also needed to be carried out. Moreover, the cases and controls in this meta-analysis were mostly based on Han nationality, but not in the minorities. In order to get comprehensive results of the Chinese population, studies based on the minorities are also needed.

There are several limitations in this meta-analysis. First, the quantity of enrolled published studies was not very ideal, especially for the -318C/T and CT60 polymorphism. This might cause some potential publication bias, although the results of the above mentioned bias tests was not significant for these two polymorphisms. Second, data were not stratified into subgroups according to some other factors such as age, gender, location and ethnicity (Han or others), due to the lack of information in the original studies. Third, the interactions between genetic factors and environmental factors were not discussed for these three polymorphisms. Fourth, the current meta-analysis only investigated the three most widely studied polymorphisms, and some other polymorphisms with fewer reports were not included. And in future, if there were more case–control studies, new meta-analysis should be conducted. Despite of these limitations, we have minimized the bias through the whole process based on means in study identification, data selection and statistical analysis as well as in the control of publication bias and sensitivity, and got a more reliable result.

## Conclusions

To our knowledge, this is the first comprehensive genetic meta-analysis performed in Chinese population for Graves’ disease and *CTLA-4* gene. We found that three polymorphisms (+49A/G, -318C/T and CT60) in the *CTLA-4* gene were associated with the risk of GD. Our results supported the classic view that GD is associated with heredity and revealed that genes in the pathogenesis are important for GD. These results may have implications for further medicine researches about GD for the Chinese population. In future, more large-scale case–control studies are needed to validate our results.

## Competing interests

The authors declare that they have no competing interests.

## Authors’ contributions

LD designed the research. JH and JQY searched the publications, extracted the data and wrote the article. YGZ checked all data. JCH and ZPX was responsible for data synthesis and helped designed the study’s analytic strategy. YGZ and LD edited the manuscript. YXM, TYX and HCW revised the manuscript. All authors read and approved the final manuscript.

## Pre-publication history

The pre-publication history for this paper can be accessed here:

http://www.biomedcentral.com/1471-2350/14/46/prepub
